# 3D virtual reconstruction and quantitative assessment of the human intervertebral disc’s annulus fibrosus: a DTI tractography study

**DOI:** 10.1038/s41598-021-86334-8

**Published:** 2021-03-25

**Authors:** Dan Stein, Yaniv Assaf, Gali Dar, Haim Cohen, Viviane Slon, Einat Kedar, Bahaa Medlej, Janan Abbas, Ori Hay, Daniel Barazany, Israel Hershkovitz

**Affiliations:** 1 Department of Anatomy and Anthropology, Sackler Faculty of Medicine, Tel Aviv University, 69978 Tel Aviv, Israel; 2 The Shmunis Family Anthropology Institute, Dan David Center for Human Evolution and Biohistory Research, Tel Aviv University, 69978 Tel Aviv, Israel; 3 Department of Neurobiochemistry, Faculty of Life Sciences, Tel Aviv University, 69978 Tel Aviv, Israel; 4 Department of Physical Therapy, Faculty of Social Welfare and Health Studies, Haifa University, Mount Carmel, 31905 Haifa, Israel; 5 Adelson School of Medicine, Ariel University, Kiryat Hamada 3, 40700 Ariel, Israel

**Keywords:** Cartilage, Anatomy, Evolution, Evolutionary developmental biology, Imaging techniques

## Abstract

The intervertebral disc’s (IVD) annulus fibrosus (AF) retains the hydrostatic pressure of the nucleus pulposus (NP), controls the range of motion, and maintains the integrity of the motion segment. The microstructure of the AF is not yet fully understood and quantitative characterization is lacking, leaving a caveat in modern medicine’s ability to prevent and treat disc failure (e.g., disc herniation). In this study, we show a reconstruction of the 3D microstructure of the fibers that constitute the AF via MRI diffusion tensor imaging (DTI) followed by fiber tracking. A quantitative analysis presents an anisotropic structure with significant architectural differences among the annuli along the width of the fibrous belt. These findings indicate that the outer annuli's construction reinforces the IVD while providing a sufficient degree of motion. Our findings also suggest an increased role of the outer annuli in IVD nourishment.

## Introduction

The intervertebral disc (IVD) plays a key role in human locomotion and is often involved in the onset of low back pain. However, its microstructure is not fundamentally understood; hence it is important to obtain its 3D quantitative representation.

The IVD is composed of two distinct regions that counter balance each other to withstand high compressive axial forces. The annulus fibrosus (AF) is arranged in concentric cylindrical layers of collagen bundles (lamellae) around the gelatinous nucleus pulposus (NP)^1,2^. The annular rings are arranged in alternating directions anchored to and beyond the adjacent vertebra’s bony epiphyseal ring^3,4^ as well as to its cartilage endplate^5^. Morphological variations in the structure most likely account for the inhomogeneity of its strength. The fibro-cartilaginous bands have been characterized, mainly via 2D histological images, and have been shown to present both circumferential and radial structural variations. Each lamella is composed of 15 to 25 collagen fiber bundles; this varies among individuals, lumbar levels, and individual IVDs, both circumferentially and radially^6^. Lamellae bands do not form complete rings because length and thickness variations cause local irregularities by merging and splitting^6,7^. The frequency of layer interruption peaks at the posterior-outer location of the annulus; lamellae density within the IVD region is reported to be lower outerly. The thickness of the lamellae also varies both radially and circumferentially and was reported to increase with age^6,8,9^. AF macro and microstructure have been studied via histology, X-ray diffraction (e.g.,^10^), light microscopy (e.g.,^10,11^), DIC microscopy^12,13^, confocal microscopy^14^, and atomic force microscopy^15^. These modalities provide little spatial or quantitative information and often require prior chemical manipulations (e.g., dehydration) that affect the sample’s morphology.

Diffusion tensor imaging (DTI) is a well-established magnetic resonance imaging (MRI) method that exploits the random movement of water molecules through living biological tissues utilizing diffusion weighted imaging (DWI). The three-dimensional properties of water diffusion enable DTI to reveal the anatomical microstructure, and to assess structures quantitatively and non-destructively, dictated by the morphology and anisotropy of the tissue^16^. Objective parameters derived from the principle diffusion directions include the indices of fractional anisotropy (FA) and mean diffusivity (MD)^17,18^. DTI was developed for imaging neural tracts in the central nervous system^18,19^ and very good correlation of anatomical detail between DTI and histology has been reported^9,20^. In recent years the use of DTI has extended much beyond the central nerve system^21–24^. Early use of DTI for characterization of porcine AF anisotropy and microstructure has been reported by Hsu and Setton^25^ and more recently, Tourell et al.^26^ have published a pilot study investigating the lamellar structure of ovine excised IVD samples with DTI.

Fiber tracking is a procedure that utilizes DWI data in order to compute the most likely pathways of water diffusion through the sample scanned. The pathways computed via fiber tracking thus represent anatomical microstructures because the diffusion process randomly occurs through three-dimensional space and is restricted only by physical variations in the tissue. The fibers computed can deliver morphometric as well as physiological parameters to produce structural information (e.g., fiber orientations). Thus, the fibers as referred to in this work, represent fibrillar structures rather than single collagen bundles. A pilot study performed with a high field unit has supported the hypothesis that tracts found via DTI tractography correspond to actual fibrillary structures^27^.

DTI and tractography, if combined with clinical data, could potentially bridge the gap between clinical symptoms and radiological findings. Therefore, the aim of this work was to establish an MRI approach to visually assess the three-dimensional structure of human AF, and equally important, to provide quantitative assessment tools for determining the structure of the AF.

In this work, we investigated the feasibility of using DTI followed by fiber tracking to anatomically view and characterize the human AF three-dimensionally as well as to quantitatively characterize the spatial variations of the mean fibers’ length, their inclination angles, the number of fibers throughout the sample, and the diffusion-related parameters (MD, FA, and individual eigenvalues). Eight individuals’ IVDs were excised in order to achieve high-resolution images and to characterize the diffusion properties of the disc at a high quality.

## Results

An analysis of the data will be presented both qualitatively and quantitatively as follows: (a) qualitative and quantitative DTI data; (b) fiber tracking data—a comparison between the grouped properties of the inner, middle, and outer annuli; and (c) a comparison of the quantitative fiber tracking data of individual samples.

### DTI qualitative analysis

**Figure 1:**
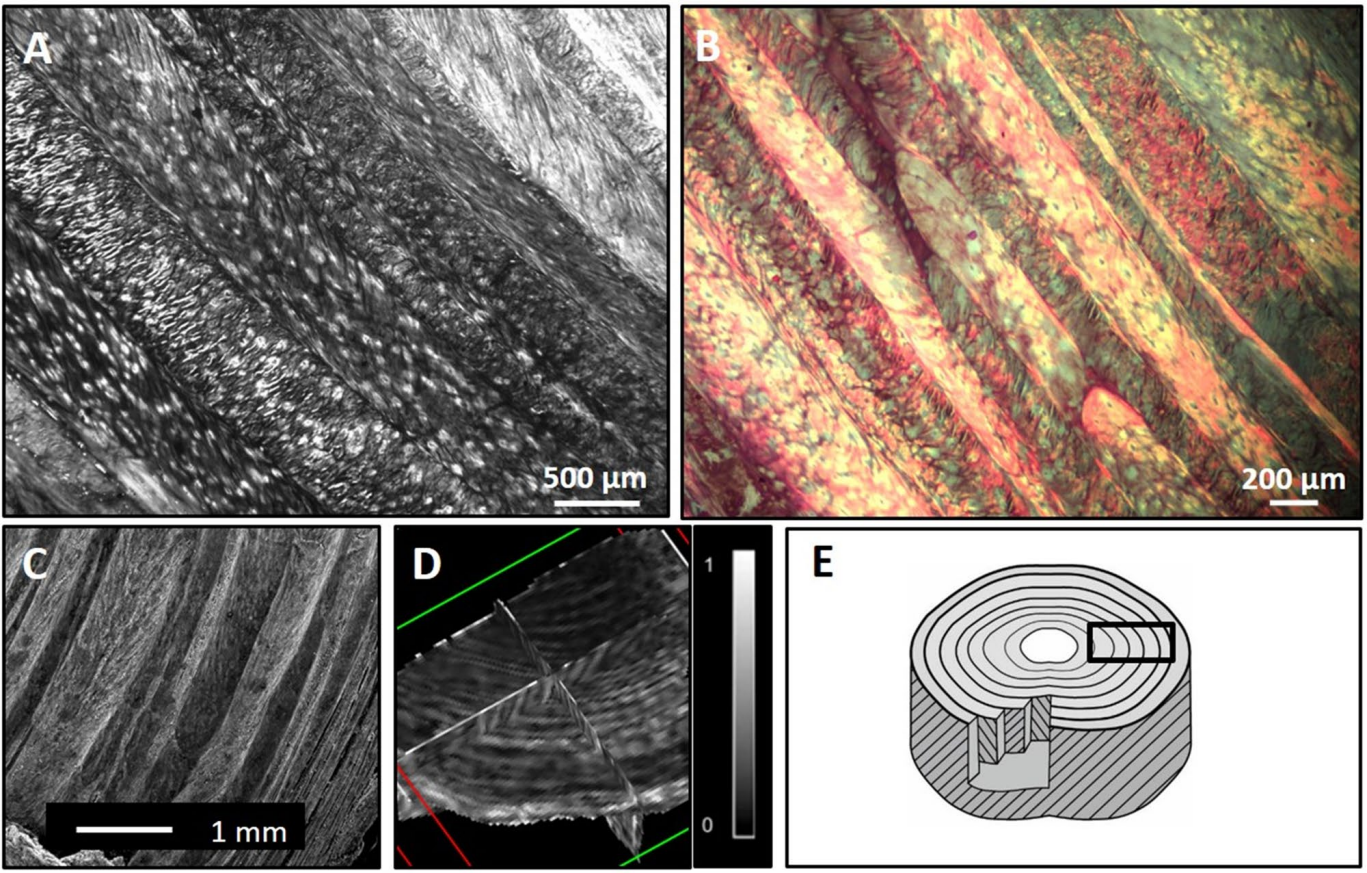
A portion of the outer AF and its structure. Two-dimensional high-resolution histological images of the AF (axial section) using light microscopy (**A**,** B**): The different orientations of the fiber tracts in each lamella are clearly seen in (**A**), whereas (**B**) (Masson Trichrome stain) presents the complex structure of the AF. The 2D scanning microscope image yielded a consistent arrangement (**C**). DTI generated 3D FA data where distinct concentric lamellae rings are seen (as in the histological sections), since they are associated with anisotropic water diffusion (**D**). The scheme (**E**) shows lamellae layers with alternating orientation fibers surrounding the nucleus pulposus (center).

For each sample, the details of the laminated AF can be seen on FA maps due to its relatively high anisotropy. These match well with the structure of the lamellae as determined by 2D histology and by 2D scanning microscope image (Fig. 1A–C). A 3D reconstruction of one sample is shown in Fig. 1D. The differences in the observed diffusion anisotropy exemplify the structural variation throughout the non-uniform lamellae width.

### DTI quantitative analysis

DTI-derived indices, which provide structural information are presented on the left side of Table 1. The FA was relatively high for DTI data given the collagenous, heterogeneous medium: 0.12–0.33 with an MD of 0.5–1.9 (× 10^−3^mm^2^/s) and total means of 0.24 ± 0.03 and 1.3 ± 0.15 (× 10^−3^mm^2^/s), respectively. The signal-to-noise ratio (SNR) for all samples was at least 28, reflecting the high quality of the scans.

**Table 1:** Quantitative DTI parameters with standard deviation from whole tissue and from tractography.

Whole tissue parameters				Tractography parameters			
Sample	FA	MD (× 10^–3^ mm^2^/s)	SNR	Axial inclination angle (°)	Mean length (mm)	Mean fiber tracts FA	Mean fiber tracts MD
1	0.12 ± 0.01	0.5 ± 0.1	44	*34*	*10* ± *7*	*0.4* ± *0.1*	*1.4* ± *0.3*
2	0.27 ± 0.03	1.9 ± 0.1	20	*37*	*4.6* ± *2.5*	*0.34* ± *0.1*	*1.3* ± *0.2*
3	0.21 ± 0.04	1.1 ± 0.2	40	*39*	*8* ± *5*	*0.39* ± *0.1*	*1.4* ± *0.2*
4	0.32 ± 0.03	1.2 ± 0.2	36	*27*	*7* ± *5*	*0.41* ± *0.1*	*1* ± *0.1*
5	0.27 ± 0.03	1.5 ± 0.1	38	*37*	*4* ± *2*	*0.43* ± *0.1*	*1.2* ± *0.2*
6	0.21 ± 0.03	1.6 ± 0.1	28	*34*	*6* ± *2*	*0.37* ± *0.1*	*1.2* ± *0.2*
7	0.15 ± 0.02	0.9 ± 0.3	30	*31*	*9* ± *7*	*0.26* ± *0.1*	*1.3* ± *0.2*
8	0.33 ± 0.03	1.7 ± 0.1	35	*34*	*6* ± *4*	*0.42* ± *0.1*	*1.3* ± *0.2*
Mean	0.24 ± 0.03	1.3 ± 0.15	34 ± 8	*34* ± *3.6*	*6.9* ± *4.4*	*0.38* ± *0.06*	*1.26* ± *0.4*

In italics, mean parameters extracted from tractography of whole samples, with standard deviations shown when applicable.

### Fiber tracking qualitative analysis

**Figure 2:**
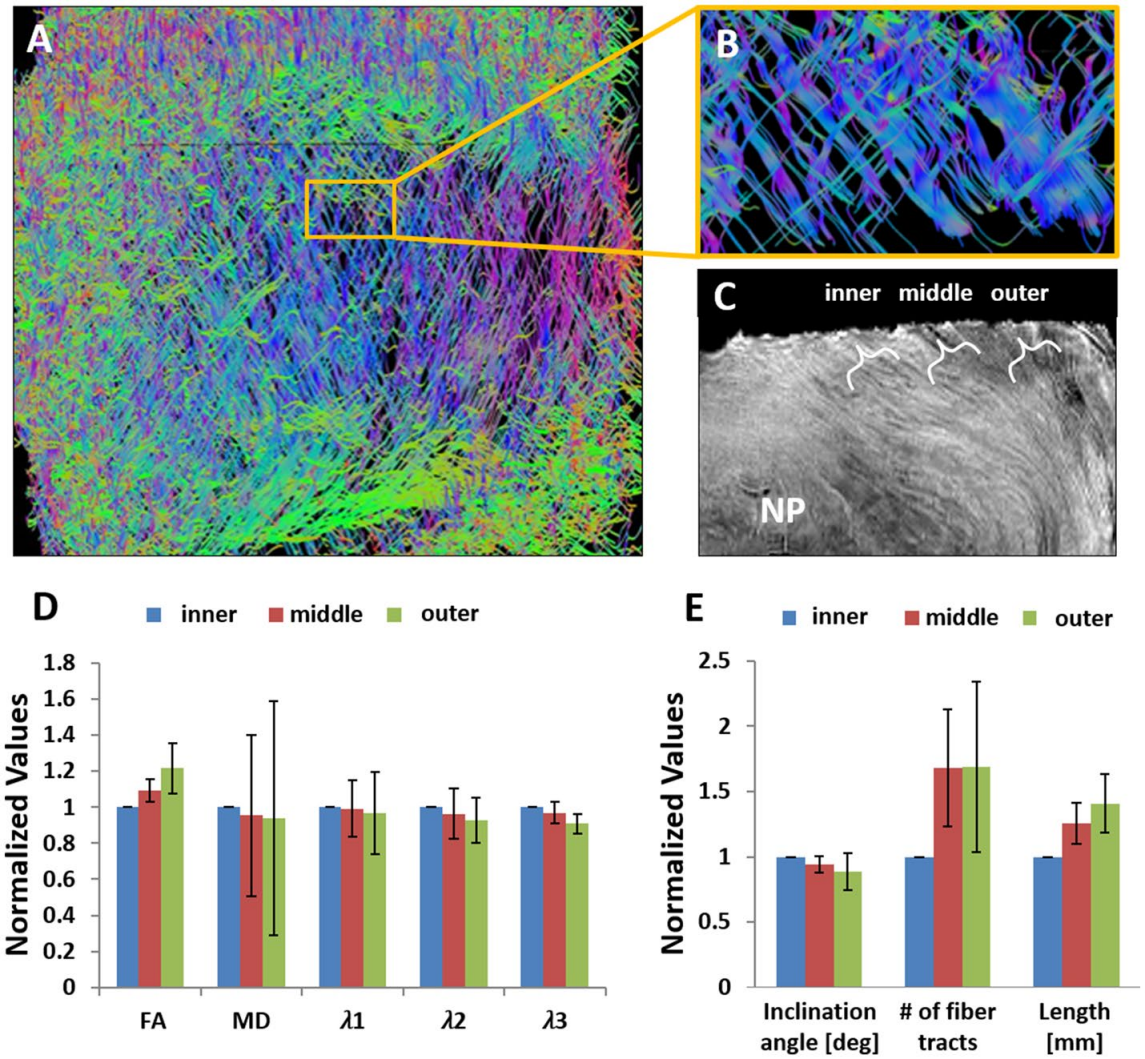
Structure of the AF. (**A**) A dense fiber tracts network typical of the 3D disc reconstruction via DTI followed by fiber tracking. For visualization purpose only 1 in 30 fiber tracts is displayed. (**B**) An enlargement of the yellow square showing parallel layers of the lamellae displaying the expected structure of crossing fiber tracts in alternating directions composing the AF. Large variations are apparent among the fiber tracts thickness, length density, and orientation. The divison into the three anatomical regions can be seen on the T2 weighted image (**C**). Normalized diffusion parameters analyzed by anatomical region with standard deviation (**D**). FA increases significantly between the inner and both the middle (p < 0.05) and outer (p < 0.001) regions (Kruskal–Wallis test). MD, λ1, and λ2 do not show significant variations between the three regions. λ3 is the only diffusion value showing a significant difference between the inner and outer region (p < 0.01). The mean morphological parameters analyzed by region (n = 8) with standard deviation (**E**). Owing to the large variations between samples, we normalized the parameters to the inner region. The inner region differs significantly from the middle and outer regions regarding the inclination angle, the number of fiber tracts, and in the tracts' length (Kruskal–Wallis test). The inclination angle shows a decreasing trend from the inner to the outer aspect of the AF, and a significant difference between both the inner and middle (p = 0.001) and the outer (p < 0.05) regions. The number of fiber tracts differs significantly between the inner and both the middle (p = 0.001) and the outer (p = 0.005) regions. A clear trend can also be seen as the mean fiber tract length increases towards the outer portion of the AF, and a significant difference is seen between the inner and both the middle (p < 0.005) and outer (p < 0.001) regions of the AF.

A detailed 3D image of the AF could be presented using fiber tracking (Fig. 2A, B). The laminated structure of the AF reveals fiber tracts that cross each other in alternating directions and that vary considerably in thickness, spatial density, and inclination angle throughout each sample and between the samples (see Movie S1 for a video clip of the 3D structure in the supplementary).

### Quantitative analysis by regions across samples

Virtual radial division has been chosen due to its correspondence to the natural symmetry of the IVD. Within each sample, the diffusion parameters (“Diffusion parameters” section) and the morphological parameters (“Morphological parameters” section), both measured from a very large fiber population (number of tracts > 10,000), contributed to the significant differences found between annuli of different regions.

#### Diffusion parameters

The mean diffusion parameters along the fiber tracts in the three anatomical regions (Fig. 2C) analyzed are shown in Table 2. The FA increased from a mean of 0.34 at the inner annuli to 0.41 at the mean outer region, whereas the MD showed an opposite, decreasing, trend towards the peripheral region. Individual diffusion vector components (λ1 is the diffusion parallel to the fibers, λ2 and λ3 are perpendicularly oriented elements of diffusion) show a similar trend, a decrease towards the peripheral annuli. When normalized to the inner aspect, the FA differs significantly when comparing the inner aspect and either the middle or the outer ones. The third eigenvalue differs significantly between the inner and outer regions (Fig. 2D).

**Table 2:** Analysis of the mean diffusion parameters along the fiber tracts with standard deviation according to circumferential regions based on their proximity to the center.

Region	FA	MD*	λ1*	λ2*	λ3*	Inclination angle (°)	No. of fiber tracts ^†^	Mean length (mm)
Inner	0.34 ± 0.06	1.34 ± 0.11	1.59 ± 0.09	1.3 ± 0.09	1.08 ± 0.10	*36.25* ± *3.11*	*37* ± *18*	*4.48* ± *0.96*
Middle	0.37 ± 0.05	1.28 ± 0.15	1.58 ± 0.15	1.25 ± 0.14	1.04 ± 0.13	*34.00* ± *2.60*	*55-* ± *19*	*5.56* ± *1.19*
Outer	0.41 ± 0.06	1.25 ± 0.10	1.54 ± 0.15	1.20 ± 0.12	0.98 ± 0.10	*31.88* ± *4.68*	*54-* ± *17*	*6.29* ± *1.76*

In italics, absolute values analysis of the mean morphological parameters (n = 8) with standard deviation divided into circumferential regions based on their proximity to the center.

*(× 10^–3^ mm^2^/s).

^†^ × 10^3^.

#### Morphological parameters

The properties of the inner, middle and outer annuli of the three parameters tested (the inclination angle, number of fiber tracts, and mean fiber tract length) show a non-significant trend among the three regions analyzed (Table 2). The fiber tracts’ inclination angles decrease by 4° from a mean of 36° in the inner annuli to 32° in the outer annuli. The number of fiber tracts detected and their length, both show an increasing trend of approximately 50% from the inner to the outer annuli. When the properties of the individual samples are normalized to the inner region and then grouped and compared, a significant difference appears between the inner region compared to either the middle or the outer regions (p < 0.05 for all differences) (Fig. 2E).

### Analysis of individual samples

**Figure 3:**
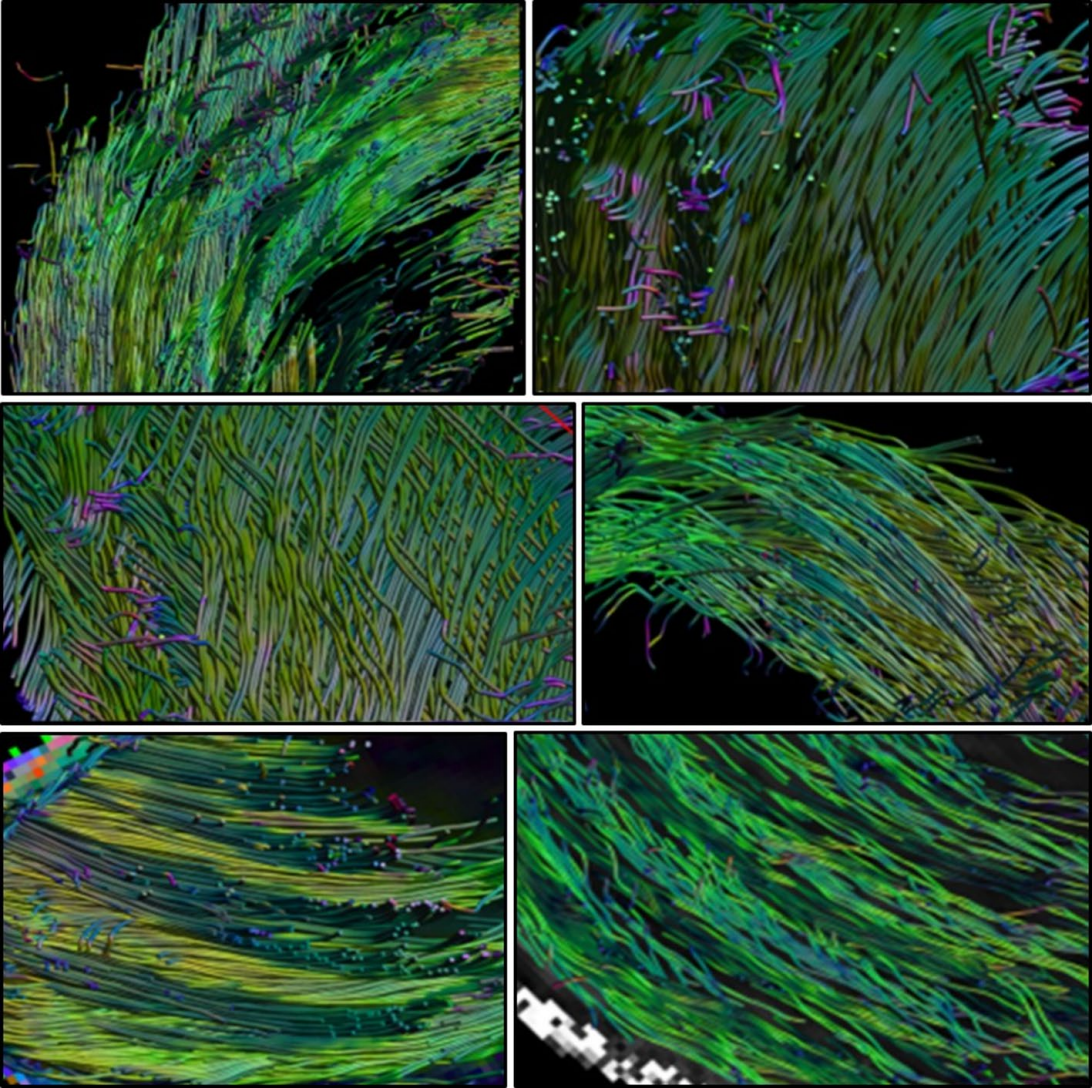
Images taken from the 3D rotating reconstruction of the fiber tracts that compose the AF in six different samples. The characteristic structure of the AF is present in all the samples scanned with local variations appearing in each image.

3D images of the samples scanned were produced (Fig. 3). Each sample contains a very high number of fiber tracts (> 10^4^), contributing to the significant differences (p < 0.001) between all tested parameters. The mean tractography extracted values for each of the samples are shown on the right side of Table 1 and the variations among the three regions were tested as follows for each parameter.

#### FA, MD, and diffusion components

**Figure 4:**
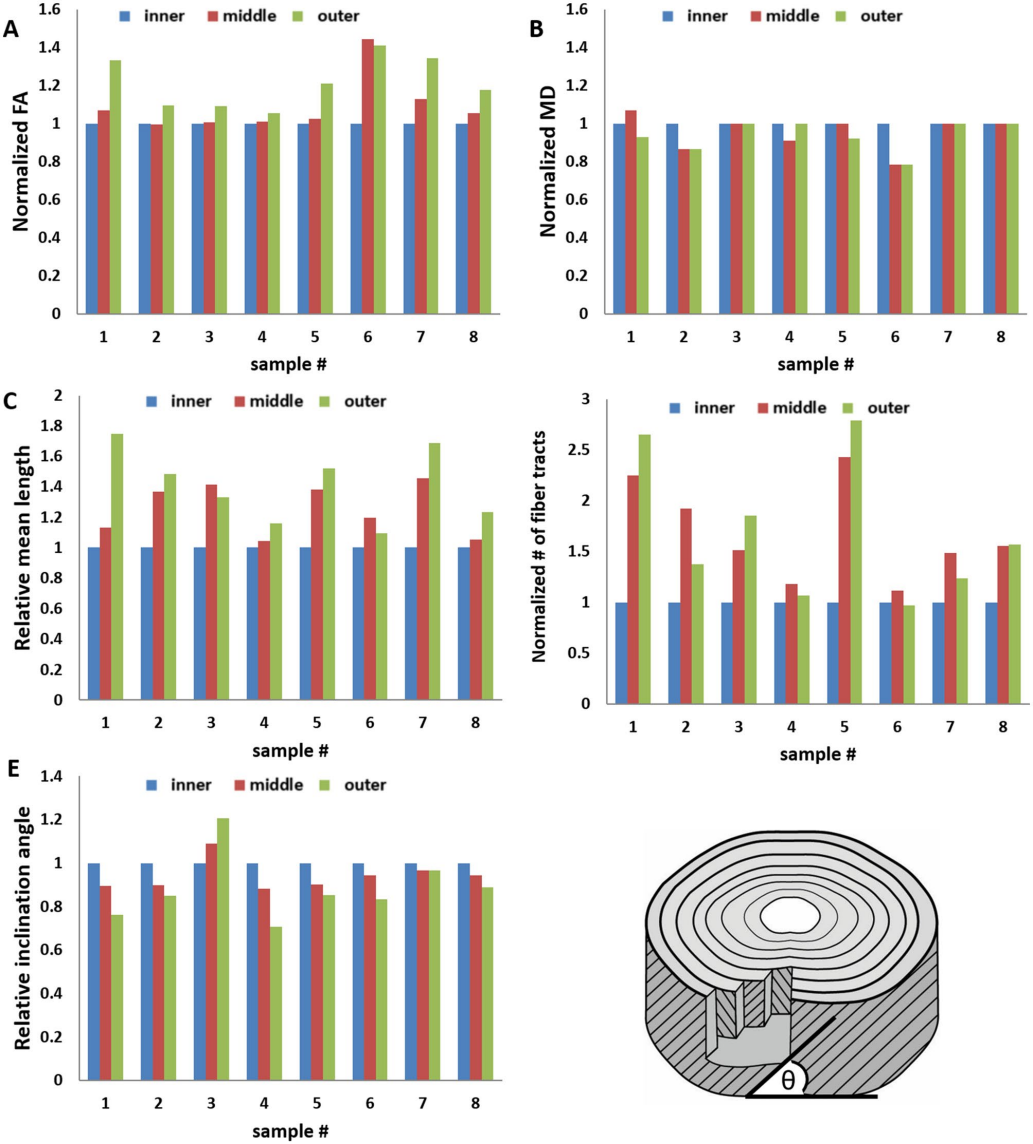
Diffusion and morphological parameters for the eight samples, normalized to the inner aspect. FA tends to increase from the inner to the outer region (**A**), whereas MD shows only a little variation among the regions (**B**). The mean fiber tracts’ length in each of the three regions after normalization to the length of the inner region (**C**). The inner region has the lowest fiber tract length throughout all the samples. In six out of the eight samples, the outer aspect of the disc has the largest mean fiber tract length. The number of fiber tracts found is higher in the outer region in four samples and is higher in the middle region in four other samples (**D**). The peaks of the axial inclination angle to the axial plane from the disc surface follow a steady pattern in seven out of the eight samples scanned, in which the highest peak appears in the inner region and decreases from there toward the outer aspects of the disc (**E**). P value < 0.001 between any two regions tested for each sample (t test), apart from MD differences, which did not show significant variations between regions.

Mean fiber tracts' FA for all samples was within the range of 0.26 ± 0.1 to 0.43 ± 0.1, with an average of 0.4 ± 0.1 (Table 1)*.* In the radial analysis, the mean FA displayed a repeating pattern of low FA on the inner part, which increases by up to 40% in the outer aspect of the AF (Fig. 4A). The mean tracts' MD for all samples was 1.4 ± 0.3 × 10^−3^mm^2^/s. MD did not present significant variations among the three regions analyzed (Fig. 4B).

#### Mean fiber tracts' length and the number of fiber tracts detected

The mean fiber tracts' length for each sample was between 4 ± 2 mm and 10 ± 7 mm, with a mean sample of 7 ± 4 mm. Normalized to the inner annuli, six of the eight samples revealed that the outer annuli had the longest fiber tract length, whereas in the two remaining samples, the middle annuli had the longest fiber tract length (Fig. 4C). The number of fiber tracts varied among the samples, probably due to the samples’ physical conditions and subtle changes in the data analysis that occurred individually for each sample (see Methods). Thus, the relative number of fiber tracts found within each sample is more informative than the absolute numbers (Fig. 4D). Across all eight samples, the number of fiber tracts found is lowest in the inner section. Four of the samples display the highest number of fiber tracts on the outer aspect, whereas the other four display the highest number in the middle region.

#### Fiber tracts' angle of inclination to the axial plane

The fiber tracts’ inclination angles to the axial plane for all eight samples analyzed were in the range of 27° to 39° with a mean of 34° ± 3.8° (Table 1). In the radial analysis of the AF, the fiber tracts' mean inclination angles were highest in the inner annuli, and then decreased from that point on towards the outerannuli, by as much as 30%. Only one sample showed an opposite pattern, with the highest inclination angle in the outer annuli and the lowest in the inner ones (Fig. 4E).

## Discussion

In this study, we used DTI to characterize the 3D morphology of collagen fibers in IVDs. Over the past two decades, the combination of DTI with fiber tracking has been used intensively in neuroscience, answering central questions while raising new ones. Recent technological advancements have enabled us to overcome technical challenges and to apply this method to the IVD.

### DTI of the AF

FA reconstructions of the AF correlate very well with the histological images due to the visibly distinct lamella layers (Fig. 1). Each lamella layer is separated from its neighbor by a thin low anisotropic layer. Thus, the laminated structure directs water diffusion movement within the disc. The mean FA values for all samples of the DTI data (measured over the entire sample without region-specific analysis via fiber tracking) were between 0.12 and 0.33 (Table 1). The FA values of the IVD have been seldom reported thus far. Zhang et al.^28^ performed in vivo DTI on volunteer subjects, achieving a much lower spatial resolution and reported FA values of 0.1–0.23. Despite these differences, the values in both studies are within a very close range. Tourell et al.^26^ have reported FA values of 0.05–0.15 in ovine specimens with large std. To rule out changes due to the use of Formaldehyde, we scanned fresh and preserved AF tissue and found no significant difference between them. Nevertheless, differences in FA values could be a result of many factors, including initial imaging parameters, technical set-up, preparation and the specimen's age. An interesting possibility is that the higher FA in our study may result from a species difference; one possibility is that the human's upright stance has altered the micro-structure of the AF to better withstand the vertical strains and to increase axial rotation; however, this hypothesis must be further tested in future studies. Articular cartilage, which is also constructed mainly from collagen but is more rigid than fibrocartilage, has been reported to show relatively low FA values of 0.02–0.41^29–31^. The FA found in the IVD is relatively low, compared with that of the central nervous system’s white matter; it is complex and less structurally ordered. FA is particularly sensitive to noise; thus, the high signal-to-noise ratio (SNR) (> 28) is an important factor reflecting low bias in the quantitative assessment. The MD in our samples was in the range of 0.5–1.9 (${x10}^{-3}{mm}^{2}$/*sec*) with a total mean of 1.3 ± 0.15 (× 10^−3^mm^2^/s) (Table 1), comparable to the reported Apparent Diffusion Coefficient (ADC) of porcine AF reported by Hsu & Setton^25^: 1.20 ± 0.05 (× 10^−3^mm^2^/s).

### Presenting a 3D reconstruction of the AF microstructure

Fiber tracking enabled us to generate three-dimensional images of the pathways by which water diffuses through the AF. The tracts found are thought to represent the orientation of the complex laminated fiber network composed of ca. 10% elastin, but mainly of type I and type II collagen, consisting of microfibrils with a zig-zag wave formation that creates a crimp. Trans-lamellar bridges have also been reported^12,13,32^, bridging adjacent lamellae. This heterogeneous array is manifested in fiber tracking, which measures the displacement of water molecules. The main pathways that constitute the AF are composed of collagen; however, these paths unfold within the different fiber types and structural variations. The three-dimensional image constructed via fiber tracking (Fig. 2A-B) can be viewed at any specific region in the sample by choosing to analyze only the fibers that pass through a selected region of interest, viewing the fibers either based on specific fiber angles from the data pool, or based on the entire 3D sample by rotating it 360° and revealing the qualitative assessment of a highly complex yet ordered structure.

### Quantitative parameters

Our results suggest that the outer annuli generally have the lowest inclination angles and contain more and longer fibrillar structures with higher FA. This heterogeneity within the AF's structure may be responsible for its reported biomechanical properties, suggesting that the AF acts nonlinearly as a composite material to shear and tensile strains^33^; that seems to reflect the collagen’s requirement for increased tensile deformations^34^, as part of a ‘smart’ cylinder^35^. Experiments seem to suggest that under high loads, the tangent modulus (E) differs between the middle annuli and the inner or outer annuli; a phenomenon that is most likely due to the anatomical variations throughout the AF^33^. Thus, our findings may be correlated both with the gross anatomical structure and at the cellular level, as was demonstrated by Bruehlmann^36^, because the three annular regions are occupied by three distinct cell populations.

### Diffusion parameters along the tracts

To the best of our knowledge, this the first study to report FA values of fiber tracts of fibrocartilage. The mean FA in the samples scanned was ~ 0.4 (Table 2). This is a moderately high FA that can be explained by the arrangement of collagen fiber bundles, and compares with the brain's neuronal white matter. Whole sample DTI sets results (FA of 0.12–0.33) (Table 1) showed significantly lower FA values due to averaging-out of the data within these samples, as opposed to the minimum threshold FA for the tract algorithm. Moreover, in this study we used extreme spatial resolution; thus, when homogeneous parts of the collagen arrangement are sampled, high anisotropy levels could be detected. The FA values generated in this study imply that relatively small differences exist between the three main eigenvalues, especially between the parallel and perpendicular components, suggesting that water diffusion is directional throughout the complex structure. Like articular hyaline cartilage, fibrocartilage is nurtured solely via diffusion; this might explain the need for highly directional pathways throughout the AF. The regional analysis shows a significant increase in FA in all samples towards the periphery by as much as 40% (Fig. 4A). The inner part of the AF gradually changes because of the gelatinous nature of the NP; thus, the AF is expected to exhibit less anisotropy. The combination of an increased FA with lower MD (Fig. 4B), along with lower values for all three main diffusion eigenvalues (λ1-λ3) in the periphery of the AF, implies that although the outer annuli are less hydrated, diffusion pathways are more distinct and in general, the region is characterized by a more ordered structure. The ratio between the components of λ1, which is the main, fast, parallel diffusion direction to the average of the perpendicular (slower) directions (λ2 and λ3), is highest in the outer annuli, explaining why this part of the AF has an overall higher anisotropy, with a higher FA.

### Fiber inclination angle

The inclination angles of the fibrillary structure directly influence the biomechanical performance of the AF. The spatial orientation of the lamellae determines the disc’s strength and stability through a wide range of movements. In our study each sample scanned showed a wide range of inclination angles, with 34° ± 3.8° (all samples combined) falling within previously determined ranges^6,9,10,37^. Previous reports also described a decrease in the values of the fiber angle from the periphery (~ 62°) of the disc inwards (~ 45°); this variation may be as high as 35°^6,11,25^. We found that the circumferential fibers of the AF are inclined to the axial vertebral plane at an angle that has a large range, of a mean ~ 27°–~ 39°, with an average of approximately 34°. Contrary to what has been previously reported, we have found the inclination angle of the outer (outer) (~32°) annuli to be significantly lower than that of the fibers in the inner annuli (~ 36°) by up to 30%, meaning that the fibers in the outer annuli are more horizontally oriented compared with those of the inner annuli. The large variation in the angles of the fibers may account for the differences found among reports.

### Number of fiber tracts

The fiber tract count is a function of many factors, including hydration state, lamellar organization, and tissue anisotropy, as well as a function of the parameters chosen for the fiber tracking analysis. For this reason, our result focuses on the relative number of fiber tracts within each sample, by normalizing them to the inner region. Because the center of the IVD is gelatinous in nature and since there is a gradual change from the NP to the AF, between which the borders are not always clear, the inner aspect of all samples contains the lowest number of fiber tracts from the three regions. Large differences between the inner, middle, or outer annuli are seen as the number of fiber tracts is reduced in the inner annuli, compared with the outer annuli by ~ 10–~ 65%. The mean normalized number of fiber tracts, from all the samples combined, is reduced by ~ 30% from the periphery towards the center (Fig. 2E). The degree of disc degeneration, which has been shown to affect quantitative diffusion parameters^37^, perhaps explains the highly variable fiber count, which may thus serve as a landmark of important clinical value.

### Fiber tracts' length

The mean fiber tracts' length found was in the range of 4–10 mm (Table 1), reflecting an increase of 10%-80% (normalized) from the inner to the outer annuli, with a mean of approximately 30% (from 4.48 ± 0.96 in the inner annuli to 6.29 ± 1.76 in the outer annuli) (Table 2). Although the fiber length reported represents the fibrillary structures rather than individual collagen bundles, our findings are in line with the report by Holzapfel et al.^35^. Longer outer fibers can be expected from the disc’s geometrical structure because the outer lamellae have longer paths to travel due to the longer outer diameter of the disc. Thus, the outer annuli present both significantly more fibers as well as longer fibers than the inner annuli. Additionally, Cassidy and Baer showed that the crimp angle of the collagen increases from ~ 20° in the outer AF to ~ 45° in the inner area of the AF; thus, the outer annuli have the least amount of crimp, resulting in very stretched fibers that are highly susceptible to tears^11^. The natural dehydration process associated with aging, which leads to a loss of disc height, may act to shorten the length of the AF fibers. Nevertheless, the outer annuli are the most common region that suffers from annular tears that lead to disc herniation. In order to cope with the rotational movements required by the bipedal stance, it is plausible that the human AF has stretched its outer lamellae to their maximum possible length, which leaves only minimal crimp angles for elongation due to rotational movements. This compromise may lower the ability of the disc to provide movement (its primary goal) for the benefit of adding strength to the annular fibers, which are in a state of maximum elongation and are on the brink of tearing.

### Outer annuli as a nutritional pathway

Owing to our upright stance, the human IVD has evolved under unique strains in order to increase the range of motion for each spinal segment. Consequently, it has grown to be among the largest in terms of absolute height and volume^38^, forcing higher nutritional demands, but at what cost? Since it is the largest avascular tissue, the human IVD is highly dependent on diffusion pathways for its nourishment and waste removal. Our results suggest that the outer annuli, located on the outer aspect of the AF, have higher anisotropy, i.e., a more ordered structure. This can be seen by examining the individual eigenvalues that contribute to the overall diffusion (Table 2). Moreover, when comparing the radial to the circumferential diffusivity along the radial axes of the AF (Fig. s2), it is evident that the higher FA in the outer part is primarily due to a reduction in circumferential diffusivity with only a small relative change in the radial diffusivity between the inner and outer AF. This has also been shown in histological studies^39^. Since the IVD receives part of its nourishment via small blood vessels penetrating only the outer lamellae^40^, we postulated that the ordered structure of the AF is well fitted for directional displacement of nutrients. If this hypothesis is true, then the role of the outer annuli as a nutrient pathway is expected to increase with advanced aging, a process associated with dehydration of the NP from reduced nutrient transport via the vertebral endplate, as the outer annuli compensate for the dehydration of the NP. It remains to be further tested whether the mechanism leading to discogenic pathologies (i.e., disc herniation, disc protrusion) stems from the onset of disc dehydration which alters the volume of nutrient flow through the outer annuli.

### Limitations of the study

This study reports the successful use of DTI, followed by fiber tracking to explore the anatomical details and to carry out a quantitative analysis of the AF. The group sampled was limited to cadavers available in the university’s dissection room; therefore, we were limited with regard to the selection of gender, age, and ethnicity. A larger population, spanning various ages and pathological conditions, could have produced a wider range of results and enabled us to follow the studied parameters across various phases of life. Moreover, cutting into the structure of the AF could have led to some changes in the fibers’ architecture; however, the desire to attain high resolution required scanning portions of the AF rather than the entire IVD. It would be highly valuable to view an intact sample, provided the technological means would allow it.

Lamellae thickness has been reported to range from approximately 100–> 500 µm and to increase with age^6,8,25^. Our samples were taken from elderly individuals, aged > 60; thus, a lamellae thickness of > 300 µm was expected to have some variation. Since our scanning resolution was approximately 225 µm, we estimated that our scans would account for most of the anatomical details. However, lamellae that are less than 225 µm might split within a single voxel and go undetected. Using an extreme resolution could facilitate the morphological analysis; however, it would also come at a cost of lower SNR in our given unit, thus being less suitable for our study.

Lastly, all samples were taken from cadavers soaked in formalin embalming solution. The use of preserved samples for scanning proved to be advantageous for performing DTI due to their rigidity, unlike fresh samples, which yielded unsuccessful results. The drawback of using samples preserved in formalin is that this solution is known to reduce the level of MD.

## Conclusions

In this study, we demonstrated the feasibility of performing DTI, followed by fiber-tracking to the human AF. We have introduced a significant step forward in better understanding the very basic building blocks of the spine by viewing the 3D structure of the AF. This has enhanced our understanding of this complex structure, shedding light on variations within different parts and across individuals, and allowing us to explore its mechanical properties. In addition, original quantitative data have been presented. If these methods are developed into an *in vivo* study, IVD can be quantitatively characterized objectively. An approach such as this could make a profound difference in early detection of pathological conditions that lead to low back pain. Early detection may provide the time necessary to plan a preventative strategy that can reduce the probability of developing this pathology. In addition, quantitative assessment of the IVD may contribute to a better understanding of the correlation between the radiological findings and the clinical symptoms.

Our study applied advanced DTI techniques and is therefore novel in three aspects: (1) It is among the first to perform DTI with fiber tracking of the AF, hence adding substantial data to the literature. (2) It is the first to present quantitative DTI indices data along the fiber tracts. (3) It is the first study to introduce a 3D visual reconstruction of the AF in humans with the use of DTI.

## Materials and methods

### Study design

This study was a controlled laboratory experiment. The sample size was not predefined and instead was determined by the availability of good-quality specimens. Inclusion criteria included samples from lumbar spines of cadavers of both sexes. Exclusion criteria included any visible spinal pathology. In addition, the technically demanding preparation of each sample for scanning (see details below) resulted in excluding approximately half of the prepared samples.

Samples: The IVD samples were taken from human cadavers at the Department of Anatomy and Anthropology, Sackler Faculty of Medicine, Tel Aviv University, by certified personnel. Informed consent to participate was obtained from the LAR of the cadavers. According to the anatomical donation program at the University of Tel Aviv, all donors signed a bequeath form of their body for the purpose of scientific and medical research. The study and the experimental protocols were approved by the committee for approval research on cadaveric material from the department of anatomy and anthropology, Sackler Faculty of Medicine: Prof. Haim Pick, head, the Department of Anatomy and Anthropology, Sackler Faculty of Medicine, Tel Aviv University, Israel, Mrs. Rachel Oz, Administrative manager, the Department of Anatomy and Anthropology, Sackler Faculty of Medicine, Tel Aviv University, Israel.

We confirm that all methods were performed in accordance with the relevant guidelines and regulations of the department. Eight IVDs were harvested from lumbar spines of human cadavers of eight individuals of both sexes of age > 60 at the time of their death. The IVDs were extracted while maintaining a thin bony surface that anchored the annular fibers to the adjacent vertebrae above and below. Each IVD was cut into segments of 10 mm^3^ and one segment from each IVD was then tightly inserted into a 15 ml plastic tube; this minimized swelling due to hydration. Though various degrees of disc degeneration were present, each sample consisted of a carefully chosen portion of the disc with no degenerative signs visible to the naked eye.

#### Preparation

Samples of the AF were kept in a 5% formaldehyde solution. MRI studies carried out on preserved brain tissue with formaldehyde show no effect of formalin fixation on anisotropy (e.g., Sun et al.^41^). Previous experiments performed in our laboratory with fixation methodologies suggested that the stiffness of the formaldehyde improved the scanning results. AF is a collagen-rich tissue; therefore, it is expected to exhibit strong dependence of T_2_ relaxation on tissue orientation. Nevertheless, experiments with our setup showed no influence of the samples' alignment on the extracted data. An important issue was the fixation of the samples prior to scanning. The process of obtaining fresh samples, followed by freezing them for preservation, and later pre-conditioning them before scanning to avoid excess swelling due to hydration, has been tested in our laboratory; however, this form of fixation resulted in poor scanning results. Instead, immediately before scanning, Fluorinert liquid was inserted into the tube with the samples in order to minimize susceptibility-induced artifacts at boundary areas as shown by Neufeld et al.^42^.

#### Imaging

All samples were imaged on a 7T/30 MRI scanner (Bruker, Germany) equipped with a quad coil fitted with the 15 ml diameter tube. MRI protocol includes a DTI protocol acquired with a diffusion-weighted spin-echo echo-planar-imaging (EPI) pulse sequence with the following parameters: TR/TE = 4000/20 ms, Δ/δ = 10/3.5 ms, 4 EPI segments, and 60 non-collinear gradient directions with a b value of 500 s/mm^2^. Geometrical parameters: 34 slices with a cubic resolution of 0.225 × 0.225 × 0.225 mm^3^. The total acquisition time was 14 h.

#### Data analysis

All data were initially analyzed via the software program ExploreDti^43^. Each scan underwent masking, motion correction, and HARDI-based DTI fiber tracking. The data were then further analyzed using custom-written Matlab (Mathworks, Inc., Natick, MA) software. Fiber tracking was performed via whole sample tensor-based stream line tractography with minimum starting FA 0.2, stopping criteria FA less than 0.1, and a maximum turning angle of 30°. From the data generated, we extracted each fiber’s length, inclination angle, FA, MD, and eigenvalues.

#### Virtual division

The IVD was virtually analyzed in accordance with its natural geometry into three circumferential sections based on the distance from the central NP: inner, middle, and outer regions (see Fig. S1 in the supplementary data). These regions were compared morphologically (the inclination angle of the fiber tracts, the number of fiber tracts found, and the mean fiber tracts' length) and by diffusion parameters: FA, MD, and individual diffusion components. Data were normalized to the inner aspect.

Research objectives: The overall goal of this study was to characterize the 3D quantitative model of the AF and to present a visual reconstruction of the fibers composing the AF using DTI followed by tractography. A pre-specified hypothesis was that morphometric measurements will be in agreement with the known findings in the literature. Post-initiation data analysis suggested that biomechanical strains drove towards a more sophisticated structure of the AF than was previously thought.

#### Statistical analyses

The nonparametric independent sample Kruskal–Wallis test was carried out using IBM SPSS (*version 20, SPSS Statistics/IBM Corp, Chicago IL, USA*) between annular regions (n = 8), whereas Students t-test was used for determining significant differences of samples within each group (n >  > 1000).

## Supplementary Information

Supplementary Legends.

Supplementary Fig. 1.

Supplementary Fig. 2.

Supplementary Video 1.
