# Supplementary material for: 3D virtual reconstruction and quantitative assessment of the human intervertebral disc’s annulus fibrosus: a DTI tractography study
Source: Sci Rep. 2021 Mar 25;11:6815. doi: 10.1038/s41598-021-86334-8 (PMC7994907; doi:10.1038/s41598-021-86334-8)
Supplement: Supplementary file 1 — Supplementary Legends. [file 41598_2021_86334_MOESM1_ESM.docx]

**3D Virtual Reconstruction and Quantitative Assessment of the Human Intervertebral Disc’s Annulus Fibrosus: a DTI Tractography Study**

Dan Stein^1^, Yaniv Assaf^2^, Gali Dar^3^, Haim Cohen^4^, Viviane Slon^1^, Einat Kedar^1^, Bahaa Medlej^1^, Janan Abbas^1^, Ori Hay^1^, Daniel Barazany^2^, Israel Hershkovitz^1^

**Affiliations**

1. Department of Anatomy and Anthropology, The Shmunis Family Anthropology Institute, Dan David Center for Human Evolution and Biohistory Research, Sackler Faculty of Medicine, Tel Aviv University, Tel Aviv, 69978, Israel.
2. Department of Neurobiochemistry, Faculty of Life Sciences, Tel Aviv University, Tel Aviv, 69978, Israel.
3. Department of Physical Therapy, Faculty of Social Welfare & Health studies, Haifa University, Mount Carmel, Haifa 31905, Israel.
4. Adelson School of Medicine, Ariel University, Kiryat Hamada 3, Ariel, 40700, Israel.

* Corresponding author: Israel Hershkovitz, e-mail: [anatom2@tauex.tau.ac.il](mailto:anatom2@tauex.tau.ac.il)

Supplementary Materials

**Fig. S1. Virtual division of the AF.** Each sample of the AF was virtually divided into an inner (blue), middle (red), and outer (green) regions, analyzed individually, and compared.

**Fig. S2. Comparison between radial and circumferential diffusion components along the radial axis.** The change in the radial diffusivity (𝜆1) compared with the change in mean circumferential diffusivity ((𝜆2+𝜆3)/2) between the inner, middle and outer locations. The values were normalized to the inner aspect, which presents the highest overall diffusivity, as it is most proximal to the nucleus pulposus. λ3 is the only diffusion value showing a significant difference between the inner and outer region (p<0.01).

**Movie S1.** A typical example of the 3D fiber orientation of the human AF as reconstructed using DTI tractography.
