# Supplementary figures and images for: 3D virtual reconstruction and quantitative assessment of the human intervertebral disc’s annulus fibrosus: a DTI tractography study
Source: Sci Rep. 2021 Mar 25;11:6815. doi: 10.1038/s41598-021-86334-8 (PMC7994907; doi:10.1038/s41598-021-86334-8)

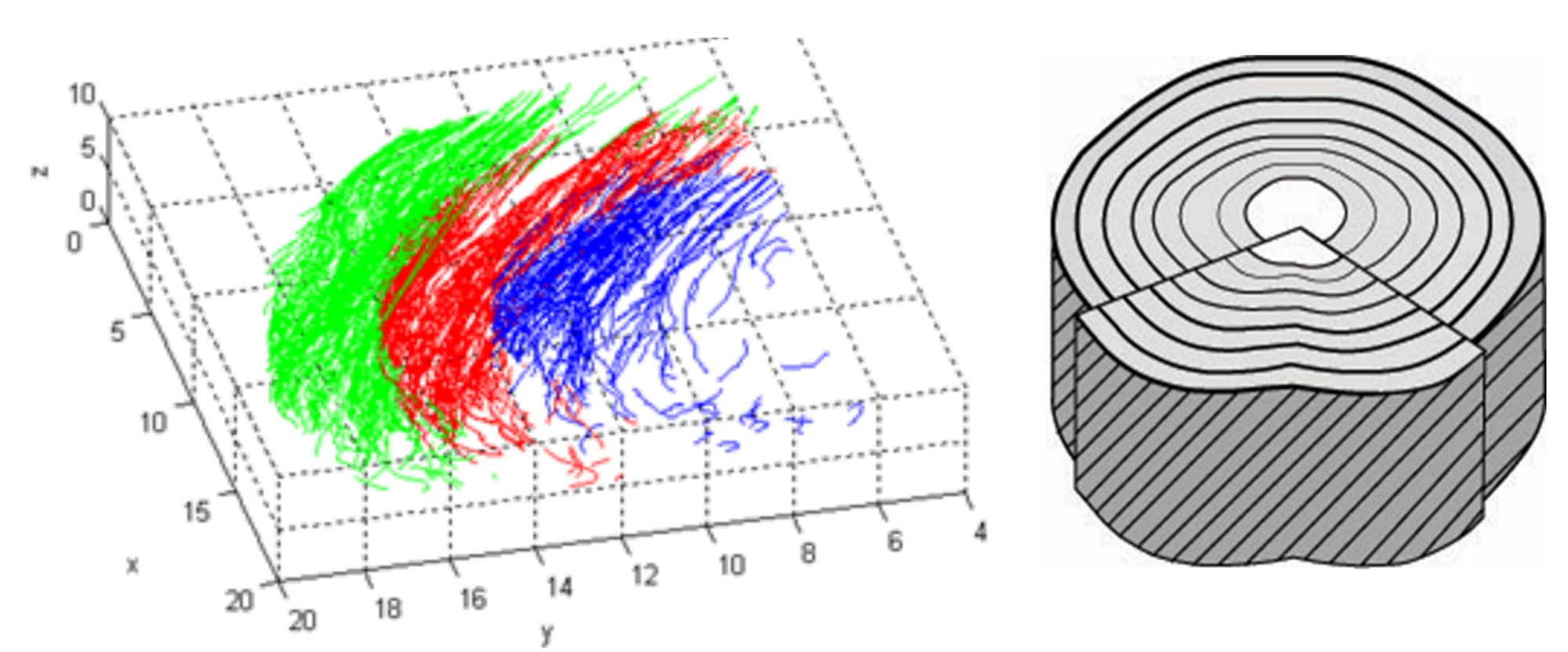

Supplement: Supplementary file 2 — Supplementary Fig. 1. [file 41598_2021_86334_MOESM2_ESM.tif]

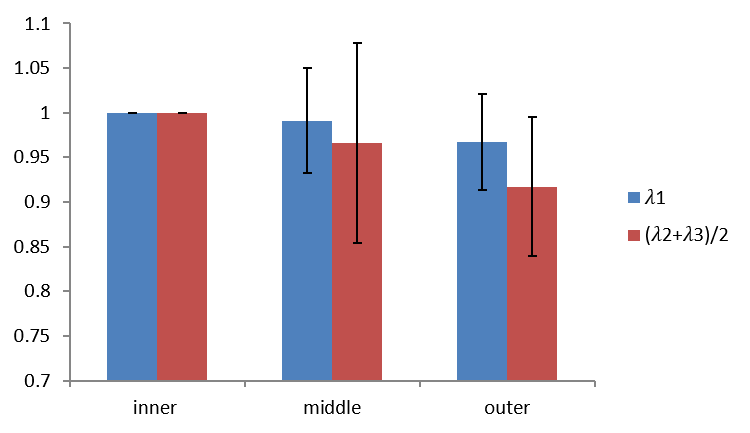

Supplement: Supplementary file 3 — Supplementary Fig. 2. [file 41598_2021_86334_MOESM3_ESM.tif]
